# Visceral adipose tissue secretome from early and late-stage oesophageal cancer patients differentially affects effector and regulatory T cells

**DOI:** 10.1007/s00432-023-04620-6

**Published:** 2023-02-15

**Authors:** Maria Davern, Dara Bracken-Clarke, Noel E. Donlon, Andrew D. Sheppard, Fiona O’ Connell, Aisling B. Heeran, Klaudia Majcher, Melissa J. Conroy, Eimear Mylod, Christine Butler, Claire Donohoe, Dearbhaile O’ Donnell, Maeve Lowery, Anshul Bhardwaj, Narayanasamy Ravi, Ashanty A. Melo, Jacintha O’ Sullivan, John V. Reynolds, Joanne Lysaght

**Affiliations:** 1 Cancer Immunology and Immunotherapy Group, Department of Surgery, School of Medicine, Trinity St. James’s Cancer Institute, Trinity Translational Medicine Institute, Dublin 8, Ireland; 2 Department of Surgery, Trinity St. James’s Cancer Institute, Trinity Translational Medicine Institute, St. James’s Hospital, Trinity College Dublin, Dublin, Ireland; 3 Department of Clinical Medicine, Trinity St. James’s Cancer Institute, Trinity Translational Medicine Institute, St. James’s Hospital, Dublin 8, Ireland

**Keywords:** TIGIT, IL-10, Ipilimumab, Nivolumab, Atezolizumab, Obesity, Oesophageal adenocarcinoma

## Abstract

**Aim:**

Visceral obesity is a key risk factor in the development of oesophagogastric junctional adenocarcinoma (OGJ), predominantly via generation of systemic low grade inflammation. Obesity-induced inflammation promotes resistance to current standards of care, enhancing tumour cell growth and survival. This study investigates the effect of the visceral adipose tissue secretome from OGJ patients with early versus advanced tumours on T-cell immunity and the role of immune checkpoint blockade in enhancing anti-tumour immunity.

**Methods and results:**

Visceral adipose conditioned media (ACM) from both early and late-stage OGJ patients significantly altered T cell activation status, upregulating co-stimulatory marker CD27 on T cells. ACM from both early and late-stage OGJ patients significantly altered immune checkpoint expression profiles downregulating immune checkpoints (ICs) on the surface of dual Th1/17-like and Th17-like cells and upregulating ICs on the surface of Th1-like cells and Treg cells. ACM derived from early-stage OGJ patients but not late-stage OGJ patients increased IFN-γ production by T cells. The addition of immune checkpoint blockers (ICBs) did not increase IFN-γ production by T cells in the presence of late-stage ACM, collectively highlighting the dichotomous immunostimulatory effect of early-stage ACM and immune-inhibitory effect of late-stage ACM. Interestingly, ACM from early-stage OGJ patients was more pro-inflammatory than ACM from late-stage patients, reflected by decreased levels of IL-17A/F, TNF-α, IL-1RA and IL-5.

**Conclusion:**

The ACM-induced upregulation of ICs on T cells highlights a therapeutic vulnerability that could be exploited by ICBs to harness anti-cancer immunity and improve clinical outcomes for OGJ patients.

**Graphical Abstract:**

Schematic workflow – (A) visceral adipose tissue was taken from OAC patients at time of surgery and cultured for 72 h in media. (B) The harvested ACM was co-cultured with healthy donor PBMCs that were concurrently activated with anti-CD3/28 for 48 h and T cell immunophenotyping was carried out by flow cytometry. Key findings – (A) Early and late stage ACM enhanced a Th1-like phenotype and upregulated CTLA-4 on Th1-like cells. A Th17-like phenotype was also enhanced in addition with a Treg-like phenotype. CTLA-4 and PD-L1 were upregulated on the surface of Treg-like cells. (B) ICB-attenuated IL-17 production by T cells. However, ACM attenuated ICB-mediated reduction in IL-10 production by T cells. Higher levels of pro-inflammatory factors were found in early stage ACM compared with late stage ACM.

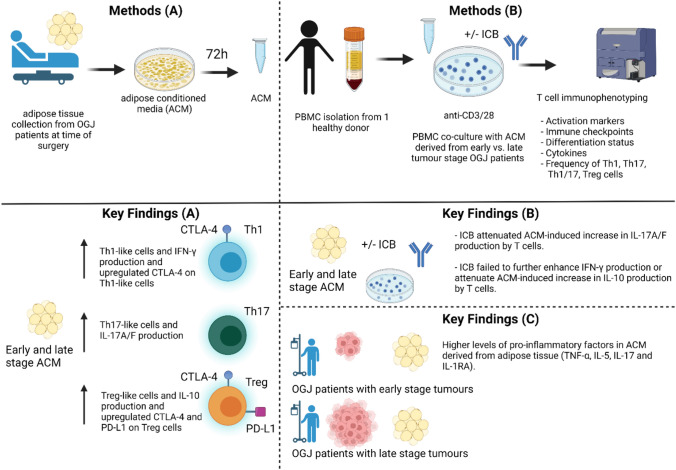

**Supplementary Information:**

The online version contains supplementary material available at 10.1007/s00432-023-04620-6.

## Introduction

It is well-established that visceral adipose tissue in oesophagogastric junctional adenocarcinoma (OGJ) patients contributes to tumour development, progression and treatment resistance via secretion of proinflammatory cytokines, insulin, fatty acids and leptin (O’Sullivan et al. 2018; Quail and Dannenberg 2019). An increase in visceral adiposity due to obesity also increases intra-abdominal pressure, forces caustic stomach and bile acids into the distal part of the oesophagus, giving rise to the pro-inflammatory condition gastroesophageal reflux disease (GORD), which affects many obese individuals and carries an increased risk of developing OGJ (Davern et al. 2021). Approximately 5% of individuals suffering with GORD progress to the pre-malignant condition of Barrett’s oesophagus (BO). It has been reported that the presence of BO carries an overall 0.05% risk of progressing to OGJ (Anaparthy and Sharma 2014). However, recent findings highlight that a risk of 0.05% may be gravely underestimating the progression rate of BO to OGJ. Nowicki-Osuch and Zhuang et al. recently demonstrated that OGJ likely arises from undifferentiated BO cell types, even in the absence of a pathologically identifiable metaplastic precursor, suggesting that the majority of OGJ pre-cursor BO lesions have gone unidentified and undiagnosed (Nowicki-Osuch et al. 2021).

Another factor that plays a key role in driving OGJ tumour initiation, progression and treatment resistance includes visceral adipose tissue via its contribution to the generation of systemic low-grade inflammation (Dumas and Brisson 2021). In contrast to lean individuals, adipocytes in obese individuals become hypertrophic, displaying an increase in size to handle increased lipid storage (Engin et al. 2019). An expansion of adipocytes leads to hypoxic areas within adipose tissue, which induces macrophage recruitment and activation, and results in the generation of a pro-inflammatory environment within adipose tissues (Divella et al. 2016; Mendonça and Soares 2015). These hypertrophic adipocytes can burst, releasing their contents into the extracellular environment. Immune cells, such as macrophages are recruited to remove the debris, however, lipids can be toxic to immune cells which induces cell death (Divella et al. 2016; Mendonça and Soares 2015). Additional immune cells, such as neutrophils are then recruited, perpetuating this state of chronic inflammation by secretion of cytokines, including interleukin (IL)-6 and tumour necrosis factor (TNF)-α which are released into the circulation generating systemic inflammation, which is pro-tumourigenic and promotes tumourigenesis at distal sites (Incio et al. 2016). IL-6 promotes anti-apoptotic pathways and TNF-α activates the transcription of oncogenes contributing to the survival of transformed cells and development of cancerous lesions (Kern 2019). OGJ patients have higher levels of circulating IL-6 in their serum compared with healthy controls and increased serum levels of IL-6 positively correlated with progression from BO to OGJ (Lin et al. 2016). IL-6 is one of the key pro-inflammatory factors produced by viscerally obese adipose tissue, playing an important role in the development of obesity-related systemic low-grade inflammation (Zhao et al. 2016). Receptors for leptin and adiponectin are upregulated in OGJ and correlate with the tumour stage and nodal involvement (Howard et al. 2010a). Leptin promotes tumour development and progression through the promotion of cell proliferation and inhibition of apoptotic pathways mediated through Akt, mitogen-activated protein kinase (MAPK), and signal transducer and activator of transcription (STAT) pathways (Park and Scherer 2011; Howard et al. 2010b).

Resistance to first-line chemo(radio)therapy regimens remains high across all solid malignancies including OGJ (Huang and Yu 2018). However, cancer immunotherapy is revolutionizing the clinical management of a variety of malignancies. In particular, immune checkpoint blockers (ICBs) targeting programmed cell death 1 (PD-1)/programmed death ligand 1 (PD-L1) pathway and targeting the cytotoxic T lymphocyte antigen-4 (CTLA-4) pathway have shown remarkable antitumor activity (Donlon 2021). Pembrolizumab and nivolumab have been FDA approved for use in OGJ (Shah et al. 2019; Joshi et al. 2018). Despite the unprecedented efficacy of ICBs, the majority of the patients do not respond to ICBs (Darvin 2018). However, high tumour mutational burden and a ‘hot’ T cell-inflamed tumour microenvironment correlate with better responses to ICB therapies (Power et al. 2020). The role of other factors, such as body mass is now also emerging as an indicator of response to ICI (Donnelly et al. 2019). In a recent study, Wang et al. (Wang et al. 2019) reported that obesity impairs T cell function characterized by increased expression of PD-1 in mice, non-human primates and humans. In addition, obesity resulted in impaired antitumor immunity and subsequently increased growth of melanoma, lung and breast tumours in mice. Leptin also upregulated the expression of PD-1 on the surface of T cells, causally linking obesity with T cell dysfunction (Wang et al. 2019). These studies highlight how obesity-induced T cell dysfunction can be exploited for cancer treatment, where the authors demonstrate that the use of ICB improved response rates in melanoma and lung tumours in obese mice and patients. Collectively these observations and findings highlight how the negative effect of obesity creates a vulnerability that can be harnessed for cancer treatment by ICBs (King et al. 2021).

It is well-known how powerful the primary tumour is in co-opting distal organs such as the visceral adipose tissue compartment, creating immunosuppressive niches and dampening systemic anti-tumour immunity. Recent reviews have highlighted the importance of adipocytes from adipose tissue in establishing a reciprocal feedback loop with tumours cells to promote cancer survival, proliferation, metastasis and treatment resistance (Dumas and Brisson 2021; Nieman et al. 1831). Cancers cells can reprogram adipocyte physiology leading to an “activated” phenotype characterized by delipidation and secretion of inflammatory adipokines such as IL-6, leptin and fatty acids which are able to change cancer cell metabolism and signalling pathways to promote tumour progression (Dumas and Brisson 2021). Therefore, for the purpose of this study we focussed on the effect of the visceral adipose tissue secretome from OGJ patients based on their tumour stage (early (pathological stage 0-II) versus late (pathological stage III-IV), on T cell phenotype and investigated whether the addition of ICBs could enhance the anti-tumour T cell phenotype.

## Methods

### Ethical approval

Ethical approval was granted from the St. James’s Hospital Ethics Committee. All samples were collected with prior informed written consent for sample and data acquisition from patients attending St. James’s Hospital or from healthy donors. This study was carried out in accordance with the World Medical Association’s Declaration of Helsinki guidelines on medical research involving human subjects. Patient samples were pseudonymised in line with GDPR and data protection policies to protect the privacy and rights of the patients.

### Specimen collection

All patients involved in this study were enrolled from 2014 to 2020. Visceral adipose tissue was obtained from OGJ patients undergoing surgical resection of tumour post-neoadjuvant treatment at St. James’s Hospital. The group consisted of 25 males and 6 females, with an average age of 66.4 years. The patient demographics are detailed in Table S1.

### Generation of adipose conditioned media

Adipose tissue conditioned media (ACM) was prepared as previously described (Conroy et al. 2016). Briefly, visceral adipose tissue was minced using a scalpel and cultured in M199 (Gibco) with 0.01% gentamicin for 72 h at 37 °C 5% CO_2_ (1 g of adipose tissue per 2 ml of media) (Kroemer et al. 2020). Following 72 h incubation the adipose conditioned media (ACM) was filtered using a 70 μM nylon mesh filter (ThermoScientific) and stored at  – 80 °C until required for experimentation. Early-stage ACM was prepared from patients whose tumours were of a pathological stage 0-II at time of surgery. Late-stage ACM was prepared from patients whose tumours were of a pathological stage III-IV.

### Cell culture

PBMCs were cultured in RPMI 1640 medium with 2 mM L-glutamine (Gibco) and supplemented with 1% (v/v) penicillin–streptomycin (50 U/ml penicillin 100 μg/ml streptomycin) and 10% (v/v) foetal bovine serum (Gibco) and maintained in a humidified chamber at 37 °C 5% CO_2_. Healthy donor PBMCs were isolated from whole blood using density gradient centrifugation and expanded with plate bound anti-CD3 (10 μg/ml, Biolegend, USA), anti-CD28 (10 μg/ml, Ancell, USA) and recombinant human IL-2 (100 units/ml, Immunotools, Germany) for 2 days in the absence and presence of ACM (diluted 1 in 2 using M199 media (Gibco) supplemented with 0.01% gentamicin with or without nivolumab (10 μg/ml), atezolizumab (10 μg/ml), ipilimumab (10 μg/ml), dual nivolumab-atezolizumab (10 μg/ml and 10 μg/ml, respectively), or dual nivolumab-ipilimumab (10 μg/ml and 10 μg/ml, respectively).

### Flow cytometry staining

PBMCs were stained with zombie aqua viability (Biolegend, USA) dye. Antibodies used for staining included CXCR3-PE, CCR6-APC, LAG-3-FITC, CD160-PerCPCy5.5, PD-1-PE/Cy7, TIGIT-PE/Cy7, CD45RA-PE/Cy7, CD45RO-BV510, CD3-APC, CD3-PerCP, CD4-BV510, CD4-APC (Biolegend, USA), CD69-PE, CD62L-FITC, CD8-BV421 (BD Biosciences, USA), CD27-APCefluor780 (eBioscience, USA), TIM-3-AF647, CTLA-4-PE/Cy5, KLRG-1-APC, PD-L1-FITC, PD-L2-PE (BD Bioscience, USA), A2aR-PE (Bio-techne, USA). PBMCs were resuspended in FACs buffer and acquired using BD FACs CANTO II (BD Biosciences) using Diva software and analysed using FlowJo v10 software (TreeStar Inc.). For intracellular cytokine staining PBMCs were treated with PMA (10 ng/ml) and ionomycin (1 µg/ml) for the last 4 h of the incubation. Anti-CD107a-PE (BD Biosciences, USA) was added during stimulation. For the last 3 h of the incubation PBMCs were treated with brefeldin A (10 µg/ml, eBiosciences). Cells were harvested, washed in FACs buffer and intracellular cytokines were assessed using a Fixation/Permeabilisation kit (BD Biosciences), as per manufacturer’s recommendations. Cells were stained with cell surface antibodies (CD8-BV421, CD3-APC or CD3-PerCP, CD4-PerCP, CD4-APC or CD4-BV510 (Biolegend, USA)) washed, permeabilised, and then stained for intracellular cytokines: IFN-γ-BV510, IL-17A-FITC, IL-10-PE and TNF-α-APC (BD Biosciences, USA). Cells were resuspended in FACs buffer and acquired using BD FACs CANTO II (BD Biosciences). For intranuclear Foxp3 transcription factor staining PBMCs were harvested, washed in FACs buffer, stained with extracellular antibodies. Using the Foxp3 Fixation/Permeabilisation kit (eBiosciences) PBMCs were fixed and permeabilised as per manufacturer’s recommendations and stained with FoxP3-BV421 (BioLegend). Cells were resuspended in FACs buffer and acquired using BD FACs CANTO II (BD Biosciences).

### Quantification of ACM secreted proteins

ACM samples were processed according to MSD (Meso Scale Discovery) multiplex protocol. To assess angiogenic, vascular injury, pro-inflammatory, cytokine and chemokine secretions a custom 54 V-plex ELISA kit and U-PLEX ELISA kit was used (Meso Scale Diagnostics, USA). The multiplex kit was used to quantify the secretions of the following analytes: CRP, Eotaxin, Eotaxin-3, FGF(basic), Flt-1, GM-CSF, IFN-γ, IL-10, IL-12p40, IL-12p70, IL-13, IL-15, IL-16, IL-17A, IL-17A/F, IL-17B, IL-1RA, IL-1α, IL-1β, IL-2, IL-21, IL-22, IL-23, IL-27, IL-31, IL-4, IL-6, IL-8, IL-9, IP-10, LAG3, MCP-1, MCP-4, MDC, MIP-1α, MIP-1β, MIP-3α, OX40, PD1, PD-L1, PD-L2, PIGF, TARC, Tie-2, TIGIT, TIM-3, TNF-α, TNF-β, TSLP, VEGF-A, VEGF-C, VEGF-D. All assays were run as per manufacturer’s recommendation, an overnight supernatant incubation protocol was used for all assays except Angiogenesis Panel 1 and Vascular Injury Panel 2 which were run according to the same day protocol. Analyte concentrations were calculated using Discovery Workbench software (version 4.0). Secretion data for all factors was normalised to weight of the adipose tissue.

### Statistical analysis

Data were analysed using GraphPad Prism version 5 (GraphPad Prism, San Diego, CA, USA) software and was expressed as mean ± SEM. Statistical differences between ACM-treated PBMCs vs untreated PBMCs were analysed using paired non-parametric *t-*test. Statistical significance was determined as *p* ≤ 0.05.

## Results

### ACM derived from OGJ patients significantly alters the expression of T cell activation and differentiation markers

**Fig. 1:**
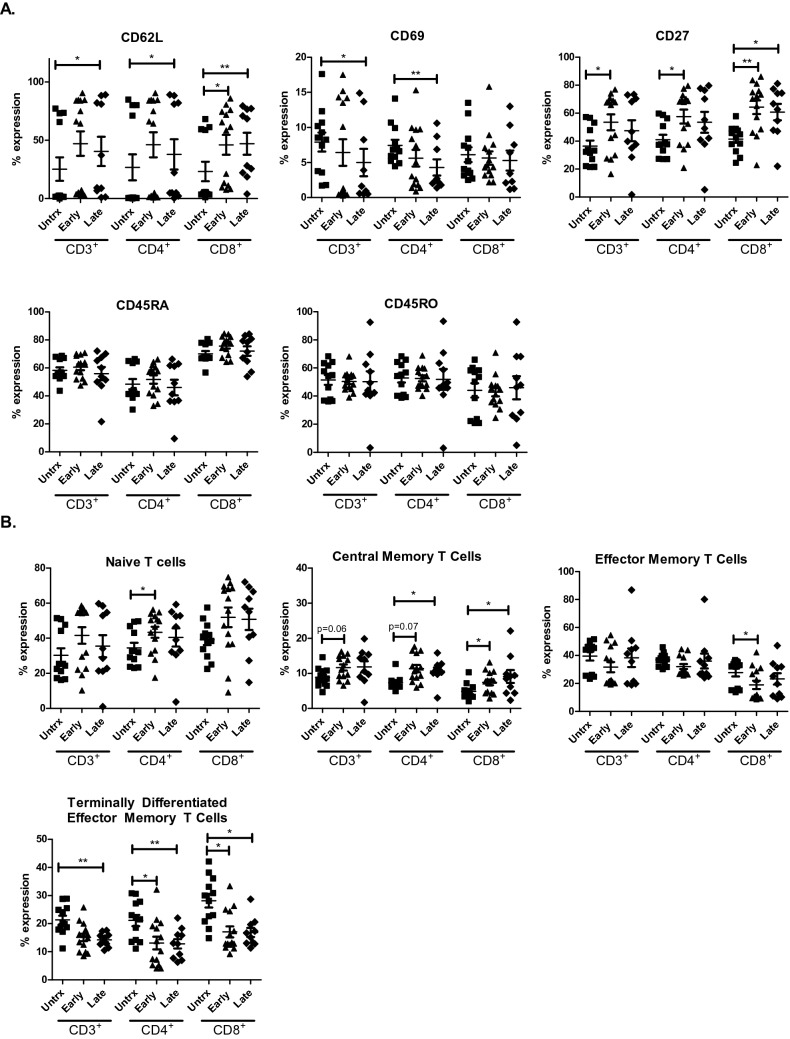
ACM derived from OGJ patients upregulates CD27 co-stimulatory marker and promotes differentiation into a central memory-like T cell phenotype. Healthy donor PBMCs were activated for 2 days using plate bound anti-CD3 and anti-CD28 in the absence (*n* = 12) or presence of ACM generated from OGJ patients with early-stage tumours (*n* = 14) or late-stage tumours (*n* = 14). CD3^+^, CD3^+^CD4^+^ and CD3^+^CD8^+^ cells were assessed for the expression of T cell activation markers: CD62L, CD69, CD27, CD45RA and CD45RO by flow cytometry (**A**). The percentage of naïve (CD45RA^+^CD27^+^), central memory (CD45RA^−^CD27^+^), effector memory (CD45RA^−^CD27^−^) and terminally differentiated effector memory (CD45RA^+^CD27^−^) T cells was also determined by flow cytometry (**B**). Paired non-parametric t test and data presented as percentages ± SEM *p < 0.05, **p < 0.01

ACM derived from OGJ patients with late-stage tumours significantly increased the expression of CD62L on the surface of CD4^+^ T cells compared with untreated cells (untrx: 26.73 ± 11.18 vs. late-stage: 37.97 ± 12.9%, *p* = 0.01) (Fig. 1A). ACM derived from OGJ patients with early-stage tumours and late-stage tumours significantly increased the expression of CD62L on the surface of CD8^+^ T cells compared with untreated cells (untrx: 23.26 ± 8.2 vs. early-stage: 46.05 ± 8.4%, *p* = 0.01, late-stage: 47.08 ± 9.3%, *p* = 0.005) (Fig. 1A). ACM derived from OGJ patients with late-stage tumours significantly decreased the expression of CD69 on the surface of CD4^+^ T cells compared with untreated cells (untrx: 7.43 ± 0.8 vs. late-stage: 4.29 ± 1.1%, *p* = 0.005) (Fig. 1A). ACM derived from OGJ patients with early-stage tumours significantly increased the expression of CD27 on the surface of CD4^+^ T cells compared with untreated cells (untrx: 41.00 ± 3.5 vs. early-stage: 57.55 ± 5.0%, *p* = 0.01) (Fig. 1A). ACM derived from OGJ patients with early-stage tumours and late-stage tumours significantly increased the expression of CD27 on the surface of CD8^+^ T cells compared with untreated cells (untrx: 41.47 ± 2.7 vs. early-stage: 64.29 ± 4.8%, p = 0.008 and late-stage: 60.75 ± 6.7%, *p* = 0.01) (Fig. 1A).

ACM derived from OGJ patients with early-stage tumours significantly increased the frequency of naïve CD4^+^ T cells compared with untreated cells (untrx: 34.53 ± 2.9 vs. early-stage: 43.39 ± 3.1%, *p* = 0.03) (Fig. 1B). ACM derived from OGJ patients with early-stage tumours and late-stage tumours increased the frequency of central memory CD4^+^ T cells compared with untreated cells (untrx: 7.35 ± 0.5 vs. early-stage: 11.39 ± 1.0, *p* = 0.07 late-stage: 10.66 ± 1.0%, *p* = 0.02) (Fig. 1B). Similarly, ACM derived from OGJ patients with early-stage and late-stage tumours significantly increased the frequency of central memory CD8^+^ T cells compared with untreated cells (untrx: 4.86 ± 0.6 vs. early-stage: 7.43 ± 0.8%, *p* = 0.05, late-stage: 9.12 ± 1.8%, *p* = 0.04) (Fig. 1B). ACM derived from OGJ patients with early-stage tumours significantly decreased the frequency of effector memory CD8^+^ T cells compared with untreated cells (untrx: 27.88 ± 2.7 vs. early-stage: 19.05 ± 3.0%, p = 0.03) (Fig. 1B). ACM derived from OGJ patients with early-stage tumours and late-stage tumours significantly decreased the frequency of terminally differentiated effector memory CD4^+^ T cells (untrx: 21.13 ± 2.0 vs. early-stage: 13.11 ± 2.2%, *p* = 0.04 and late-stage: 12.80 ± 1.6%, *p* = 0.001) and terminally differentiated effector memory CD8^+^ T cells compared with untreated cells (untrx: 28.15 ± 2.4 vs. late-stage: 17.07 ± 1.9%, *p* = 0.05, and late-stage: 16.85 ± 1.6%, *p* = 0.01) (Fig. 1B). There was no significant differences in T cell activation status or differentiation status between ACM derived from early versus late-stage OGJ patients.

### ACM derived from OGJ patients significantly alters the immune checkpoint expression profile of T cells

**Fig. 2:**
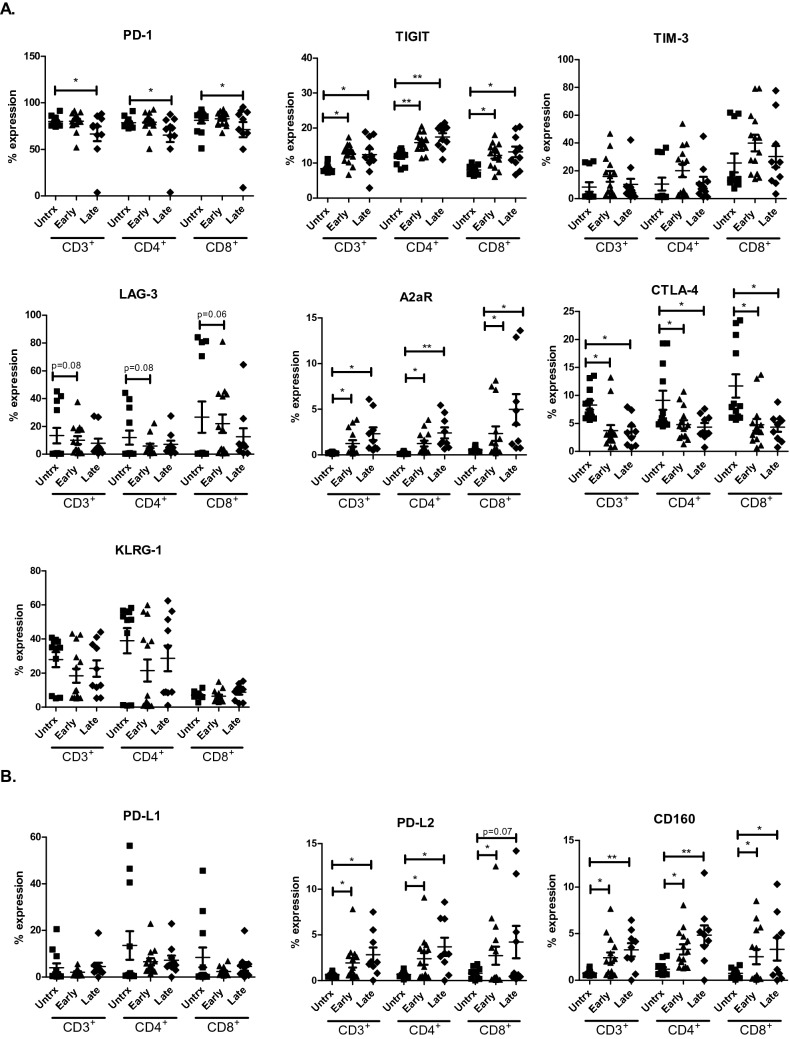
ACM derived from OGJ patients significantly upregulates TIGIT on the surface of T cells. Healthy donor PBMCs were activated for 2 days using plate bound anti-CD3 and anti-CD28 in the absence (*n* = 12) or presence of ACM generated from OGJ patients with early-stage tumours (*n* = 14) and late-stage tumours (*n* = 14). CD3^+^, CD3^+^CD4^+^ and CD3^+^CD8^+^ cells were assessed for the expression of inhibitory IC receptors including PD-1, TIGIT, TIM-3, LAG-3, A2aR, CTLA-4 and KLRG-1 (**A**) and IC ligands including PD-L1, PD-L2 and CD160 (**B**) by flow cytometry. Paired, non-parametric *t*-test, *p < 0.05, **p < 0.01

Given our findings demonstrating that ACM substantially altered the activation status of T cells, we sought to investigate if ACM also altered immune checkpoint (IC) expression profiles of T cells as this would help guide identification of appropriate ICs to target in OGJ patients. ACM derived from OGJ patients with late-stage tumours decreased the expression of PD-1 on the surface of CD4^+^ T cells (untrx: 78.63 ± 1.5 vs. late-stage: 72.32 ± 3.7%, *p* = 0.06) compared with untreated cells (Fig. 2A). ACM derived from OGJ patients with early-stage and late-stage tumours significantly increased the expression of TIGIT on the surface of CD4^+^ T cells (untrx: 11.80 ± 0.5 vs. early-stage: 15.86 ± 0.7%, *p* = 0.006, late-stage: 17.45 ± 1.0%, *p* = 0.003) and CD8^+^ T cells (untrx: 8.05 ± 0.3 vs. early-stage: 12.19 ± 0.9%, *p* = 0.02, late-stage: 13.21 ± 1.5%, *p* = 0.009) compared with untreated cells (Fig. 2A). ACM derived from OGJ patients with early-stage and late-stage tumours significantly increased the expression of A2aR on the surface of CD4^+^ T cells (untrx: 0.21 ± 0.03 vs. early-stage: 1.24 ± 0.3%, *p* = 0.01, late-stage: 2.41 ± 0.6%, *p* = 0.007) and CD8^+^ T cells (untrx: 0.54 ± 0.07 vs. early-stage: 2.33 ± 0.7%, *p* = 0.03, late-stage: 4.97 ± 1.6%, *p* = 0.02) compared with untreated cells (Fig. 2A). ACM derived from OGJ patients with early-stage and late-stage tumours significantly decreased the expression of CTLA-4 on the surface of CD4^+^ T cells (untrx: 9.12 ± 1.7 vs. early-stage: 4.80 ± 0.7%, *p* = 0.04, late-stage: 4.34 ± 0.7%, *p* = 0.01) and CD8^+^ T cells (untrx: 11.70 ± 2.0 vs. early-stage: 4.75 ± 1.0%, *p* = 0.04, late-stage: 4.33 ± 0.8%, *p* = 0.02) compared with untreated cells (Fig. 2A). ACM derived from OGJ patients with early-stage and late-stage tumours increased the expression of PD-L2 on the surface of CD4^+^ T cells (untrx: 0.65 ± 0.08 vs. early-stage: 2.38 ± 0.6%, *p* = 0.02, late-stage: 3.69 ± 0.9%, *p* = 0.01) and CD8^+^ T cells (untrx: 0.80 ± 0.1 vs. early-stage: 2.73 ± 0.9%, *p* = 0.03, late-stage: 4.21 ± 1.7%, *p* = 0.07) compared with untreated cells (Fig. 2B). ACM derived from OGJ patients with early-stage and late-stage tumours increased the expression of CD160 on the surface of CD4^+^ T cells (untrx: 1.18 ± 0.2 vs. early-stage: 3.30 ± 0.5%, *p* = 0.01, late-stage: 4.82 ± 1.1%, *p* = 0.007) and CD8^+^ T cells (untrx: 0.73 ± 0.1 vs. early-stage: 2.51 ± 0.7%, *p* = 0.01, late-stage: 3.32 ± 1.2%, *p* = 0.04) compared with untreated cells (Fig. 2B). There was no significant differences in alteration of IC expression profiles between ACM derived from early versus late-stage OGJ patients.

### ACM derived from OGJ patients promotes a Th1-like phenotype

**Fig. 3:**
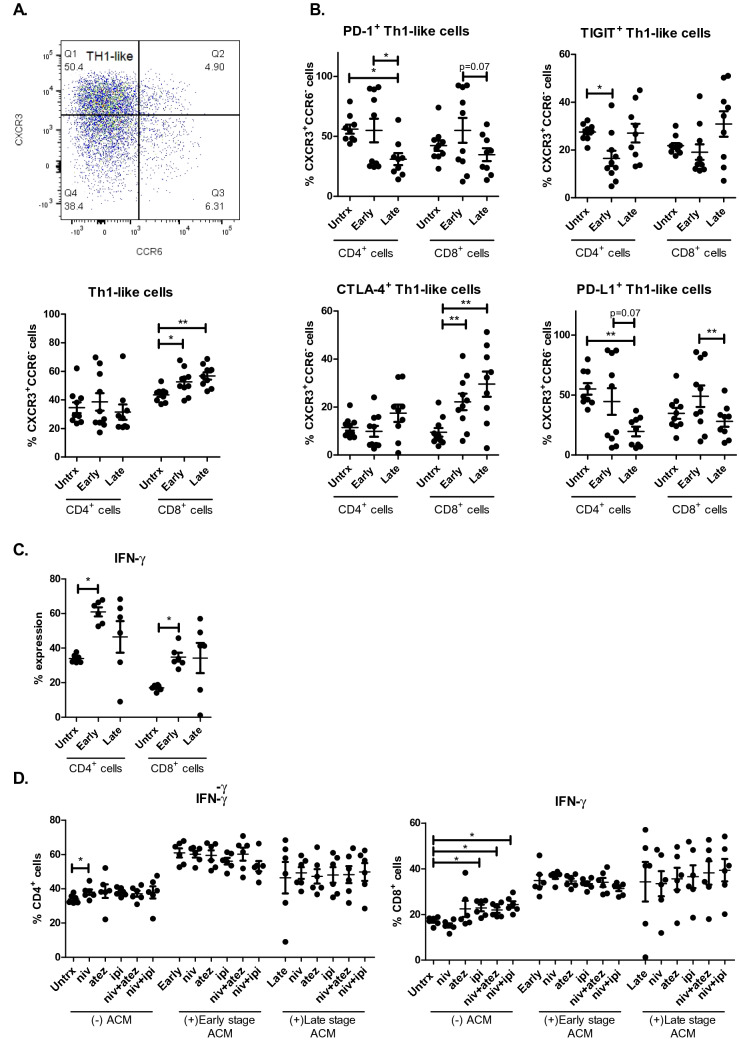
ACM derived from OGJ patients promotes a Th1-like phenotype and upregulates CTLA-4 expression on the surface of Th-1 like cells. The effect of early-stage ACM (*n* = 9) and late-stage ACM (*n* = 8) on the frequency of Th1-like CD3^+^CD4^+^ and CD3^+^CD8^+^ cells was assessed by flow cytometry and compared with untreated cells (*n* = 10). The effect of early-stage ACM (*n* = 9) and late-stage ACM (*n* = 8) on the expression of TIGIT, CTLA-4, PD-1 and PD-L1 was assessed on the surface of CD3^+^CD4^+^Th1-like and CD3^+^CD8^+^Th1-like cells by flow cytometry (**B**). The effect of ACM on the secretion of Th1-like cytokine IFN-γ was also assessed in CD3^+^CD4^+^ and CD3^+^CD8^+^ cells by flow cytometry (**C**). The effect of single agent nivolumab, single agent atezolizumab, single agent ipilimumab, dual nivolumab-atezolizumab or dual nivolumab-ipilimumab in the absence (*n* = 6) or presence of early-stage ACM (*n* = 6) and late-stage ACM (*n* = 6) on the secretion of anti-tumour cytokine IFN-γ was assessed in CD3^+^CD4^+^ and CD3^+^CD8^+^ cells by flow cytometry (**D**). Th1-like cells were characterised as CXCR3^+^CCR6^−^ cells. Representative dot plot shown displaying frequency of CD4^+^ Th1-like cells (**A**). Paired, non-parametric *t* test and data presented as percentages ± SEM. *p < 0.05 and **p < 0.01. Abbreviation: *niv* nivolumab, *atez* atezolizumab, *ipi* ipilimumab

The findings from this study demonstrated that ACM derived from OGJ patients significantly altered T cell activation status and IC expression profiles. Therefore, we sought to investigate if ACM might alter an anti-tumour Th1-like phenotype which plays a key role orchestrating anti-tumour immunity in OGJ. ACM derived from OGJ patients with early-stage tumours and late-stage tumours significantly increased the frequency of Th1-like CD8^+^ cells compared with untreated cells (untrx: 43.65 ± 1.5 vs. early-stage: 52.64 ± 2.8 *p* = 0.01, and late-stage: 56.79 ± 2.6%, *p* = 0.003) (Fig. 3A). In addition, ACM significantly altered the IC expression profile of Th1-like cells. ACM derived from late-stage OGJ patients significantly decreased PD-1 expression on the surface of CD4^+^ Th1-like cells (untrx: 56.06 ± 3.7 vs. late-stage: 31.06 ± 5.0%, *p* = 0.01) compared with untreated cells (Fig. 3B). ACM derived from early-stage OGJ patients significantly decreased TIGIT expression on the surface of CD4^+^ Th1-like cells (untrx: 27.57 ± 1.0 vs. late-stage: 16.50 ± 3.2%, *p* = 0.01) compared with untreated cells (Fig. 3B). In contrast, ACM derived from early-stage and late-stage OGJ patients significantly increased CTLA-4 expression on the surface of CD8^+^ Th1-like cells (untrx: 9.47 ± 1.7 vs. early-stage: 22.13 ± 3.4%, *p* = 0.003, late-stage: 29.56 ± 5.2%, *p* = 0.007) compared with untreated cells (Fig. 3B). Furthermore, ACM derived from early-stage OGJ patients significantly increased the frequency of CD4^+^IFN-γ^+^ cells compared with untreated cells (untrx: 33.99 ± 1.0 vs. early-stage: 60.95 ± 2.6%, *p* = 0.03) and CD8^+^IFN-γ^+^ cells compared with untreated cells (untrx: 17.12 ± 0.7 vs. early-stage: 34.84 ± 2.5%, *p* = 0.03) (Fig. 3C).

We next investigated if ICB could enhance production of anti-tumour cytokine IFN-γ by T cells in the absence and presence of early and late-stage ACM to understand how the secretome of visceral adipose tissue might affect ICB efficacy in OGJ patients. Nivolumab treatment significantly increased the frequency of CD4^+^IFN-γ^+^ cells compared with untreated cells (untrx: 33.99 ± 1.0 vs. nivolumab: 38.04 ± 1.5%, *p* = 0.03) (Fig. 3D). However, nivolumab did not significantly increase the frequency of CD4^+^IFN-γ^+^ cells in the presence of ACM derived from early-stage or advanced stage OGJ patients compared with untreated cells in the presence of ACM. Single agent ipilimumab, dual nivolumab-atezolizumab and dual nivolumab-ipilimumab significantly increased the frequency of CD8^+^IFN-γ^+^ cells compared with untreated cells (untrx: 17.12 ± 0.7 vs. ipilimumab: 22.84 ± 1.4%, *p* = 0.03, nivolumab-atezolizumab: 21.93 ± 1.2%, *p* = 0.03, nivolumab-ipilimumab: 24.34 ± 1.4%, *p* = 0.03) (Fig. 3D)**.** However, ICB did not significantly affect the frequency of CD8^+^IFN-γ^+^ cells in the presence of ACM derived from early-stage or advanced stage OGJ patients compared with untreated cells in the presence of ACM.

### ACM derived from OGJ patients enhances a Th17-like phenotype; an effect which is abrogated by ICB

**Fig. 4:**
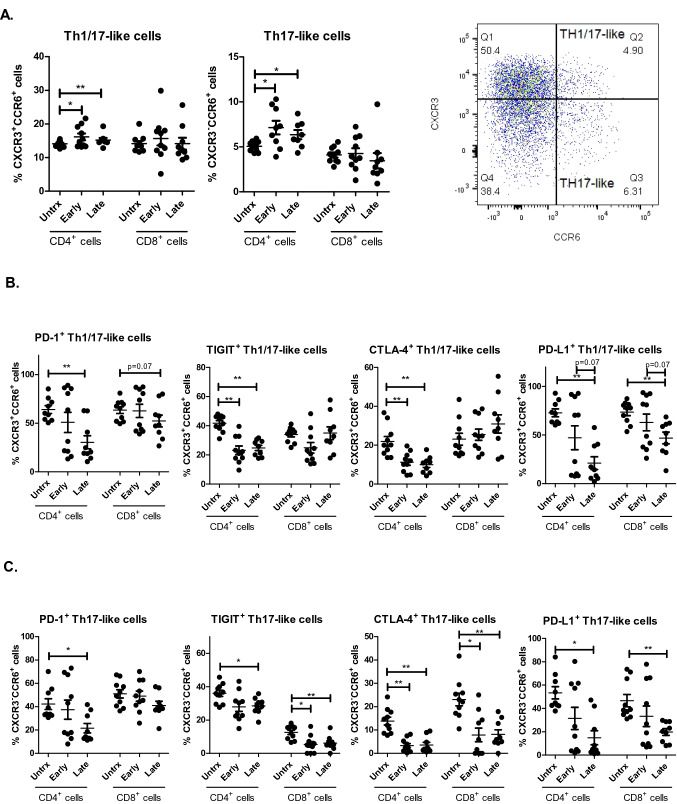
ACM derived from OGJ patients increases the frequency of pro-inflammatory-like Th-17 cells and downregulates ICs on their surface. The effect of early-stage ACM (*n* = 9) and late-stage ACM (*n* = 8) on the frequency of Th1/17-like and Th17-like CD3^+^CD4^+^ and CD3^+^ CD8^+^ cells was assessed by flow cytometry and compared with untreated cells (*n* = 10) (**A**). The effect of early-stage ACM (*n* = 9) and late-stage ACM (*n* = 8) on the expression of TIGIT, CTLA-4, PD-1 and PD-L1 was assessed on the surface of CD4^+^Th1/17-like and CD8^+^Th17-like cells (**B**) CD4^+^Th17-like cells and CD8^+^Th17-like cells (**C**) by flow cytometry and compared with untreated cells (*n* = 10). Th1/17-like cells were characterised as CXCR3^+^CCR6^+^ cells and Th17-like cells were characterised as CXCR3^−^CCR6^+^ cells. Representative dot plot shown displaying frequency of CD4^+^ Th1/17-like cells and Th17-like cells (**A**). Paired, non-parametric *t* test and data presented as percentages ± SEM. *p < 0.05 and **p < 0.01

The role of pro-inflammatory Th17 and dual Th1/17 in either promoting or hindering tumour progression remains conflicting within the literature and whether these cell types help eradicate the tumour or promote its progression is likely dependent on the tumour type and tumour-immune contexture. This study also investigated if ACM derived from OGJ patients had an effect on the frequency of Th1/17-like and Th17-like cells and the expression of pro-inflammatory IL-17A/F and TNF-α. ACM derived from OGJ patients with early-stage tumours and late-stage tumours significantly increased the frequency of Th17-like CD4^+^ cells (untrx: 5.07 ± 0.2 vs. early-stage: 7.14 ± 0.7%, *p* = 0.01, and late-stage: 6.37 ± 0.5%, *p* = 0.05) and the frequency of Th1/17-like CD4^+^ T cells (untrx: 14.07 ± 0.2 vs. early-stage: 16.15 ± 0.9%, *p* = 0.02, and late-stage: 15.19 ± 0.6%, *p* = 0.007) compared with untreated cells (Fig. 4A). ACM significantly altered the IC expression profile of Th-17-like and Th1/17-like cells downregulating PD-1, PD-L1, CTLA-4 and TIGIT surface expression (Fig. 4B and C). ACM derived from late-stage OGJ patients decreased PD-1 expression on the surface of CD4^+^Th1/17-like cells (untrx: 64.12 ± 3.9 vs. late-stage: 30.49 ± 6.7%, *p* = 0.007) and CD8^+^Th1/17-like cells (untrx: 63.51 ± 3.3 vs. late-stage: 52.34 ± 6.0%, *p* = 0.07) compared with untreated cells (Fig. 4B). ACM derived from both early-stage and late-stage OGJ patients significantly decreased TIGIT expression on the surface of CD4^+^Th1/17-like cells (untrx: 41.79 ± 1.7 vs. early-stage: 23.20 ± 2.9%, *p* = 0.002, late-stage: 24.81 ± 1.8%, *p* = 0.003) compared with untreated cells (Fig. 4B). Similarly, ACM derived from both early-stage and late-stage OGJ patients significantly decreased CTLA-4 expression on the surface of CD4^+^Th1/17-like cells (untrx: 21.98 ± 2.5 vs. early-stage: 11.28 ± 1.6%, *p* = 0.009, late-stage: 9.96 ± 1.4%, *p* = 0.003) compared with untreated cells (Fig. 4B). ACM derived from late-stage OGJ patients significantly decreased PD-L1 expression on the surface of CD4^+^Th1/17-like cells (untrx: 72.86 ± 3.9 vs. late-stage: 21.12 ± 6.5%, *p* = 0.003) and CD8^+^Th1/17-like cells (untrx: 73.52 ± 3.5 vs. late-stage: 46.97 ± 6.0%, *p* = 0.003) compared with untreated cells (Fig. 4B). ACM derived from late-stage OGJ patients significantly decreased PD-1 expression on the surface of CD4^+^Th17-like cells (untrx: 42.19 ± 4.5 vs. late-stage: 21.52 ± 4.03%, *p* = 0.02) compared with untreated cells (Fig. 4C). ACM derived from late-stage OGJ patients significantly decreased TIGIT expression on the surface of CD4^+^Th17-like cells (untrx: 36.00 ± 1.8 vs. late-stage: 28.47 ± 1.6%, *p* = 0.01) compared with untreated cells (Fig. 4C). ACM derived from early-stage and late-stage OGJ patients significantly decreased TIGIT expression on the surface of CD8^+^Th17-like cells (untrx: 12.60 ± 13 vs. early-stage: 5.37 ± 1.6%, *p* = 0.009, late-stage: 6.16 ± 1.4%, *p* = 0.002) compared with untreated cells (Fig. 4C). ACM derived from early-stage and late-stage OGJ patients significantly decreased CTLA-4 expression on the surface of CD4^+^Th17-like cells (untrx: 13.83 ± 1.7 vs. early-stage: 3.29 ± 0.9%, p = 0.002, late-stage: 3.56 ± 1.1%, *p* = 0.003) compared with untreated cells (Fig. 4C). ACM derived from late-stage OGJ patients significantly decreased PD-L1 expression on the surface of CD4^+^Th17-like cells (untrx: 53.40 ± 5.1 vs. late-stage: 14.90 ± 6.0%, *p* = 0.01) and CD8^+^Th17-like cells (untrx: 46.66 ± 5.2 vs. late-stage: 19.80 ± 2.8%, *p* = 0.003) compared with untreated cells (Fig. 4C).

**Fig. 5:**
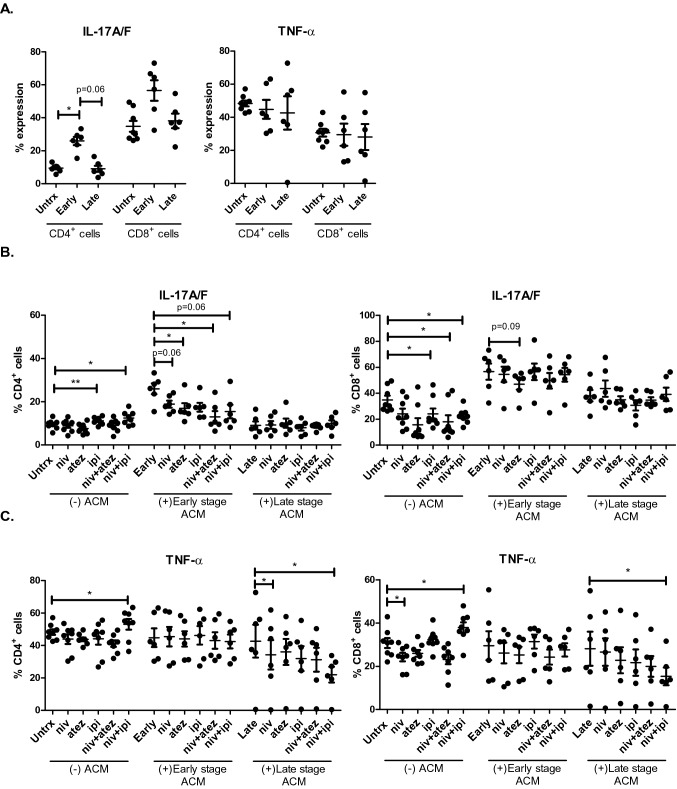
ICBs attenuates the ACM-enhanced pro-inflammatory T cell cytokine signature. The effect of early-stage (*n* = 9) ACM and late-stage ACM (*n* = 8) derived from OGJ patients on the secretion of pro-inflammatory cytokines IL-17A/F and TNF-α was assessed in CD3^+^CD4^+^ and CD3^+^CD8^+^ cells by flow cytometry and compared with untreated cells (*n* = 10) (**A**). The effect of single agent nivolumab, single agent atezolizumab, single agent ipilimumab, dual nivolumab-atezolizumab or dual nivolumab-ipilimumab in the absence (*n* = 6) or presence of early-stage ACM (*n* = 6) and late-stage ACM (*n* = 6) on the secretion of pro-inflammatory cytokines IL-17A/F (**B**) and TNF-α (**C**) was also assessed in CD3^+^CD4^+^ and CD3^+^CD8^+^ cells by flow cytometry. Paired, non-parametric t test and data presented as percentages ± SEM. *p < 0.05 and **p < 0.01. Abbreviation: *niv* nivolumab, *atez* atezolizumab, *ipi* ipilimumab

Given that ACM derived from early and late-stage OGJ patients significantly altered the frequency of Th-1/17-like and Th17-like cells and their IC expression profile, this study next sought to investigate if the ACM from these patients significantly altered the production of the pro-inflammatory cytokines IL-17A/F and TNF-α. ACM derived from early-stage OGJ patients significantly increased the frequency of IL-17-producing CD4^+^ cells compared with untreated cells (untrx: 9.49 ± 0.7 vs. early-stage: 25.95 ± 2.4%, *p* = 0.03) (Fig. 5A). ACM derived from early-stage or late-stage OGJ patients did not significantly affect the frequency of TNF-α-producing T cells compared with untreated cells (Fig. 5A). Single agent ipilimumab and dual nivolumab-ipilimumab treatment significantly increased the frequency of IL-17-producing CD4^+^ cells compared with untreated cells (untrx: 9.49 ± 0.7 vs. ipilimumab: 11.16 ± 0.5%, *p* = 0.007, nivolumab-ipilimumab: 12.47 ± 1.1%, *p* = 0.01) (Fig. 5B). In contrast, single agent nivolumab, atezolizumab, dual nivolumab-atezolizumab and dual nivolumab-ipilimumab decreased the frequency of IL-17-producing CD4^+^ cells in the presence of early-stage ACM compared with cells treated with early-stage ACM alone (early ACM: 25.95 ± 2.4 vs. early ACM + nivolumab: 18.87 ± 1.5%, *p* = 0.06, early ACM + atezolizumab: 16.95 ± 2.3%, *p* = 0.03, early ACM + nivolumab-atezolizumab: 12.96 ± 2.7%, *p* = 0.03 and early ACM + nivolumab-ipilimumab: 15.48 ± 3.1%, *p* = 0.06) (Fig. 5B). Single agent atezolizumab, dual nivolumab-atezolizumab and dual nivolumab-ipilimumab significantly decreased the frequency of IL-17-producing CD8^+^ cells compared with untreated cells (untrx: 34.83 ± 3.3 vs. atezolizumab: 15.67 ± 5.1%, *p* = 0.03, nivolumab-atezolizumab: 18.05 ± 4.9%, *p* = 0.03 and nivolumab-ipilimumab: 22.48 ± 2.1%, *p* = 0.03) (Fig. 5B). ICBs did not significantly affect the frequency of IL-17-producing CD8^+^ T cells in the presence of early-stage or late-stage ACM compared with untreated cells (Fig. 5B). Dual nivolumab-ipilimumab significantly increased the frequency of TNF-α-producing CD4^+^ cells compared with untreated cells (untrx: 48.29 ± 1.7 vs. nivolumab-ipilimumab: 53.01 ± 3.2%, *p* = 0.04) (Fig. 5C). Single agent nivolumab and dual nivolumab-ipilimumab significantly decreased the frequency of TNF-α-producing CD4^+^ cells in the presence of late-stage ACM compared with cells treated with late-stage ACM alone (late ACM: 42.65 ± 10.1 vs. late ACM + nivolumab: 34.26 ± 9.1%, *p* = 0.03, late ACM + nivolumab-ipilimumab: 21.99 ± 4.8%, *p* = 0.03) (Fig. 5C). Single agent nivolumab significantly decreased and dual nivolumab-ipilimumab significantly increased the frequency of TNF-α-producing CD8^+^ cells compared with untreated cells (untrx: 30.70 ± 2.3 vs. nivolumab: 24.26 ± 1.9%, *p* = 0.04, nivolumab-ipilimumab: 37.88 ± 2.3%, *p* = 0.05) (Fig. 5C). In contrast, dual nivolumab-ipilimumab significantly decreased the frequency of TNF-α-producing CD8^+^ cells in the presence of late-stage ACM compared with cells treated with late-stage ACM alone (late ACM: 28.10 ± 7.8 vs. late ACM + nivolumab-ipilimumab: 15.32 ± 4.1%, *p* = 0.03) (Fig. 5C).

### ACM derived from OGJ patients significantly increases the secretion of IL-10 by T cells

**Fig. 6:**
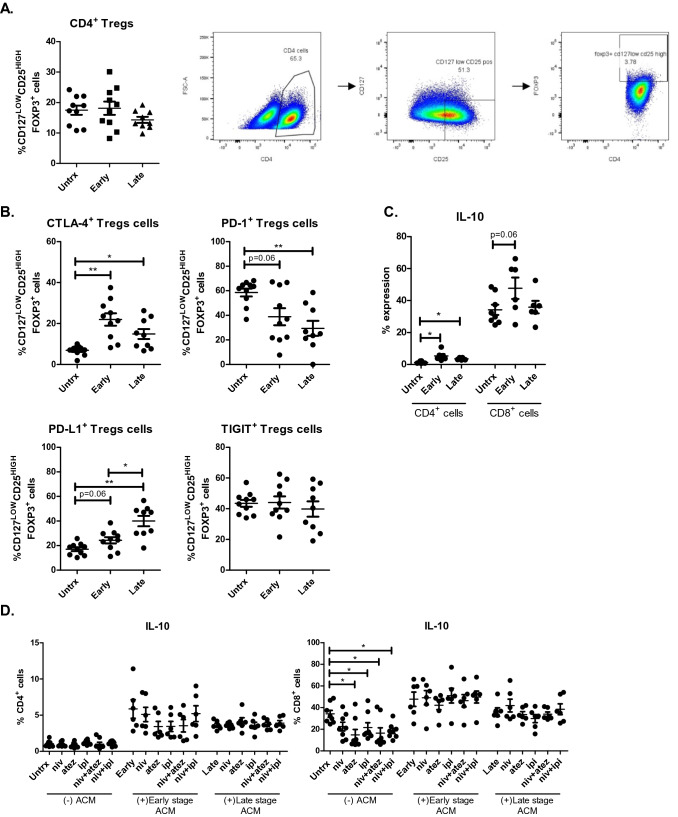
ACM derived from OGJ patients increases IL-10 production by T cells, an effect which is attenuated by ICB. The effect of early-stage ACM (*n* = 6) and late-stage ACM (*n* = 6) on the frequency of CD4^+^ Treg cells was assessed by flow cytometry (**A**). The effect of early-stage ACM (*n* = 6) and late-stage ACM (*n* = 6) on the expression of TIGIT, CTLA-4, PD-1 and PD-L1 was assessed on the surface of CD4^+^ Treg cells by flow cytometry (**B**). The effect of ACM on the secretion of anti-inflammatory regulatory cytokine IL-10 was also assessed in CD3^+^CD4^+^ and CD3^+^CD8^+^ cells by flow cytometry (**C**). The effect of single agent nivolumab, single agent atezolizumab, single agent ipilimumab, dual nivolumab-atezolizumab or dual nivolumab-ipilimumab in the absence or presence of early-stage ACM (*n* = 6) and late-stage ACM (*n* = 6) on the secretion of anti-inflammatory regulatory cytokine IL-10 was also assessed in CD3^+^, CD3^+^CD4^+^ and CD3^+^CD8^+^ cells by flow cytometry (**D**). CD4^+^ Treg cells were characterised as the frequency of CD4^+^CD127^LOW^CD25^HIGH^ that were FOXP3^+^. Representative dot plots displayed in (**A**) showing gating strategy for Treg cells. Paired, non-parametric t test and data presented as percentages ± SEM. *p < 0.05 and **p < 0.01

Regulatory T cells (Treg) play a vital role in driving tumour progression through dampening anti-tumour immune responses. It is well-established that the visceral adipose tissue is a source of pro-inflammatory mediators, however, with immune stimulation immunoregulatory processes are also activated in order to maintain homeostasis (Booth et al. 2015). Therefore, this study investigated if ACM derived from OGJ patients had an effect on the Treg compartment. ACM derived from OGJ patients with early-stage tumours and late-stage tumours did not significantly affect the frequency of CD4^+^ Treg cells compared with untreated cells (Fig. 6A). Early-stage and late-stage ACM decreased the expression of PD-1 on the surface of Treg cells compared with untreated cells (untrx: 58.57 ± 3.2 vs. early-stage: 38.86 ± 6.8%, *p* = 0.06, late-stage: 29.47 ± 6.1%, *p* = 0.007) (Fig. 6B). Early-stage and late-stage ACM significantly increased the expression of CTLA-4 on the surface of Treg cells compared with untreated cells (untrx: 6.90 ± 0.7 vs. early-stage: 21.98 ± 3.0%, *p* = 0.005, late-stage: 14.86 ± 2.4%, *p* = 0.01) (Fig. 6B), and similarly, increased the expression of PD-L1 on the surface of Treg cells compared with untreated cells (untrx: 17.07 ± 1.5 vs. early-stage: 24.29 ± 2.6%, *p* = 0.06, late-stage: 39.93 ± 4.2%, *p* = 0.003) (Fig. 6B).

Early-stage and late-stage ACM significantly increased the frequency of IL-10^+^CD4^+^ T cells compared with untreated cells (untrx: 1.28 ± 0.2 vs. early-stage: 5.37 ± 1.2%, *p* = 0.03, late-stage: 3.47 ± 0.3%, *p* = 0.03) (Fig. 6C). Similarly, early-stage ACM increased the frequency of IL-10^+^CD8^+^ T cells compared with untreated cells (untrx: 34.21 ± 3.2 vs. early-stage: 47.72 ± 6.6%, *p* = 0.06) (Fig. 6C). Single agent atezolizumab, ipilimumab, dual nivolumab-atezolizumab and dual nivolumab-ipilimumab significantly decreased the frequency of IL-10^+^CD8^+^ T cells compared with untreated cells (untrx: 34.21 ± 3.2 vs. atezolizumab: 14.82 ± 4.9%, *p* = 0.03, ipilimumab: 21.64 ± 4.42%, *p* = 0.05, nivolumab-atezolizumab: 16.29 ± 5.1%, *p* = 0.03 and nivolumab-ipilimumab: 18.47 ± 2.4%, *p* = 0.01) (Fig. 6C). However, ICBs did not significantly decrease the frequency of IL-10^+^CD8^+^ T cells in the presence of ACM compared with cells treated with ACM alone (Fig. 6C).

### ACM derived from early-stage OGJ patients is more inflammatory than ACM derived from late-stage OGJ patients

We next profiled the ACM from OGJ patients with early versus late-stage tumours to determine if there was a significant difference in the pro-inflammatory profile of the visceral fat secretome. ACM derived from OGJ patients with early-stage tumours had significantly increased levels of pro-inflammatory TNF-α compared with ACM derived from OGJ patients with late-stage tumours (early-stage: 2,730 ± 1,412 vs. late-stage: 806.1 ± 131.8 pg/gram *p* = 0.05) (Figure S1). Similarly, ACM derived from OGJ patients with early-stage tumours had increased levels of other pro-inflammatory cytokines: IL-1RA, IL5 and IL-17A compared with ACM derived from OGJ patients with late-stage tumours (IL-1RA- early-stage: 29,939 ± 13,234 vs. late-stage: 7,312 ± 1,452 pg/gram p = 0.08, IL-5- early-stage: 3.68 ± 13.98 vs. late-stage: 7.20 ± 2.5 pg/gram *p* = 0.06, IL-17A- early-stage: 62.00 ± 10.81 vs. late-stage: 40.61 ± 8.5 pg/gram *p* = 0.08) (Figure S1B–D). There was no significant difference in the levels of the remaining 49 mediators in ACM derived from early versus late-stage OGJ patients, which included immunomodulatory, pro-inflammatory and pro-angiogenic mediators shown in Figure S2.

## Discussion

As the tumour progresses, OGJ patients can start to lose weight unintentionally due to numerous factors, including changes in nutritional intake and cachexia. Due to this weight loss, the visceral adipose depot will be affected (Anandavadivelan and Lagergren 2016). Another important factor to consider is how the primary tumour will distally affect the visceral adipose tissue depot and the amplitude of this effect may likely depend on how advanced the primary tumour is. Therefore, it is plausible to hypothesise that more advanced staged tumours will have a greater immunosuppressive effect on the visceral adipose tissue compartment compared with earlier stage tumours. Therefore, this study aimed to investigate the effect of the visceral adipose tissue secretome from OGJ patients with both early- and late-stage tumours, on T cell phenotypes and whether addition of PD-1, PD-L1 or CTLA-4 ICBs might enhance an anti-tumour T cell phenotype (Pardoll 2012).

ACM from both early- and late-stage cancer patients significantly increased a range of inhibitory ICs including TIGIT, A2aR, PD-L2 and CD160 on the surface of T cells, however, there was no significant difference between early-stage or late-stage ACM. TIGIT, A2aR, PD-L2 and CD160 signalling dampens Th1 anti-tumour immunity and promotes a Treg phenotype, aiding cancer progression (Pardoll 2012). Therefore, the ACM secretome may be creating a therapeutic niche for the use of ICBs to harness anti-tumour immunity through blocking inhibitory signalling of TIGIT A2aR, PD-L2 and CD160 on the surface of T cells (Hintzen et al. 1994).

An important focus of this study was to assess the effect of ACM on IC expression profiles on anti-tumour or tumour-promoting T cells. Interestingly, we observed that ACM differentially affected the expression of ICs on Th1-like, Th1/17-like, Th17-like and Treg-like cells. Although ACM decreased CTLA-4 expression on the surface of the entire T cell population, a dichotomous effect was observed on specific T cell subsets. ACM significantly increased CTLA-4 expression on the surface of Th1-like cells and Treg-like cells, however, downregulated CTLA-4 expression on the surface of Th1/17-like cells and Th17-like cells. This highlights the importance of investigating the IC expression profile on specific T cell subsets to help guide selection of appropriate ICs to target in clinical trials. CTLA-4 plays an important role in promoting conversion of Th1 cell types into Treg cell types and enhancing Treg function (Razmara et al. 2008). Perhaps in this setting ACM may be driving the conversion of Th1-like cells to Treg cells via upregulation of CTLA-4. CTLA-4 also plays an integral role in maintaining a Treg phenotype (Razmara et al. 2008; Walker 2013). Therefore, CTLA-4 blockade may be a more appropriate IC to target for boosting Th1-like immunity in OGJ patients. In addition, ACM derived from both early- and late-stage tumours significantly increases PD-L1 expression on the surface of Treg cells, with late-stage ACM increasing PD-L1 significantly more. In contrast, ACM from both early- and late-stage OGJ patients decrease PD-1 expression on the surface of Treg cells. Amarnath et al. have indicated that PD-L1 back-signalling in T cells induces the conversion of Th1 cells into a regulatory T cell phenotype (Amarnath et al. 2011). These findings collectively highlight that targeting PD-L1 and not just PD-1 alone may be more beneficial in harnessing anti-tumour immunity in particular in the OGJ setting (Peinado et al. 2017).

ACM derived from OGJ patients with early-stage tumours and not late-stage tumours significantly increased the production of IFN-γ by T cells compared with untreated cells. This highlights the immunomodulatory and pro-inflammatory potential of the ACM derived from patients with early-stage tumours. This IFN-γ-promoting effect was not observed by late-stage ACM and highlights the immunosuppressive ability of more advanced stage tumours in co-opting distal organs to dampen their innate anti-cancer mechanisms and perhaps sculpt the visceral adipose tissue into an immunosuppressive tumour permissive niche amenable to metastatic deposition and colonisation. Although late-stage ACM did not increase IFN-γ production by T cells, the ability of ICB to enhance IFN-γ production by T cells was lost in the presence of late-stage ACM highlighting the immunosuppressive effects of the secretome of visceral adipose tissue in advanced OGJ patients.

To further understand the immunological differences in the secretome of early versus advanced ACM which may explain these differential effects on anti-tumour T cell immunity a range of immunomodulatory and tumour-promoting factors were screened in early and late-stage ACM. ACM derived from early-stage OGJ patients possessed a more inflammatory secretory phenotype. ACM derived from OGJ patients with early-stage tumours possessed higher levels of IL-5, which can have a dichotomous role in either inhibiting or promoting Th1 immunity according to the evidenced-based literature (Hung et al. 1998; Ellyard et al. 2007). IL-5 is a well-known Th2 cytokine (Koyasu and Moro 2011) and Th2 immunity has been shown to favour tumour growth via promoting angiogenesis and inhibiting anti-tumour immunity (Ellyard et al. 2007). However, there are studies demonstrating anti-tumour activity of CD4^+^Th2 cells particularly in collaboration with tumour-infiltrating granulocytes, such as eosinophils. Hung et al., demonstrated that mice deficient in IL-5 exhibited reduced anti-tumour immunity (Hung et al. 1998). Furthermore, the loss of systemic anti-tumor immunity was associated with the absence of eosinophils at the tumour challenge sites in IL-5^−/−^ mice (Hung et al. 1998). Further studies are needed to understand if higher levels of IL-5 in the visceral fat of early-stage OGJ patients may have a positive or negative effect in the context of local and systemic anti-tumour immunity in the adipose tissue compartment and also in terms of how the systemic effects of higher levels of IL-5 in the adipose tissue may affect the distal tumour (Koyasu and Moro 2011).

Higher levels of pro-inflammatory TNF-α and IL-1RA were also identified in ACM derived from OGJ patients with early-stage tumours compared with late-stage tumours. IL-1RA has been implicated in promoting tumour-associated macrophage-mediated metastasis in breast cancer (Wang et al. 2018). Additional studies have reported similar roles for IL-1RA in promoting tumour invasiveness and metastasis in other cancer types (Apte and Voronov 2017). TNF-α has been implicated in several cancer types in promoting tumour cell migration invasion and metastasis (Cruceriu et al. 2020), as well as promoting pro-tumourigenic immune cell phenotypes such as neutrophils (Vieira et al. 2009) and tumour-associated macrophages (Parameswaran and Patial 2010) and promoting resistance to current standard of care regimens via enhancing an apoptotic tumour cell resistant phenotype (Kern 2019). Collectively, this highlights that factors within the visceral adipose tissue of OGJ patients with early-stage tumours may possess a greater potential to promote tumour progression and metastasis. Further studies are required to investigate if TNF-α or IL-1RA may be playing a role in metastasis in early-stage OGJ patients and whether targeting these soluble factors might prevent metastatic dissemination in OGJ patients or prevent tumour progression to more advanced stages. However, ICBs only attenuated TNF-α production by T cells in the presence of late-stage ACM and not early-stage ACM, highlighting that the pro-inflammatory effect of ACM from early-stage OGJ patients may not be amenable to abrogation by these ICBs. Although ICB-mediated downregulation of TNF-α in the presence of late-stage ACM would be a beneficial effect to dampen tumour-promoting inflammatory responses which drive resistance to current standards of neoadjuvant care, particularly in late-stage OGJ tumours which are more resistant to chemo(radio)therapy regimens (O’Sullivan 2018). 

In addition, ACM-derived from early-stage OGJ patients and not late-stage OGJ patients significantly increased the production of pro-inflammatory IL-17 by T cells. The role of IL-17 in either promoting or dampening anti-tumour immunity has been controversial with studies reporting conflicting effects of IL-17 in either promoting or inhibiting anti-tumour immunity in different solid tumour types (Vitiello and Miller 2019). To further support this hypothesis that ACM derived from OGJ patients with early-stage tumours is promoting IL-17, higher levels of IL-17 were found in the secretome from early-stage ACM compared with late-stage ACM of OGJ patients. Lu et al*.,* identified an anti-tumour role for IL-17 in enhancing anti-tumour immunity by promoting the migration of NK cells, T cells and dendritic cells in oesophageal squamous cell carcinoma patients and in enhancing the killing capability of B cells through enhanced expression of Granzyme B and FasL (Lu 2016). In contrast, IL-17 promoted breast tumour progression via the recruitment of pro-tumorigenic neutrophils to the tumour site (Benevides et al. 2015). ICBs attenuated IL-17 production by T cells in the presence of ACM, which may be beneficial in dampening tumour-promoting inflammatory responses in the OGJ setting, given its incidence is strongly associated with obesity-driven inflammation (O’Sullivan 2018).

Collectively, this study demonstrated that ACM derived from both early and late-stage OGJ patients upregulates inhibitory ICs TIGIT, A2aR, PD-L2 and CD160 on the surface of T cells and specifically upregulates CTLA-4 on the surface of Th1-like cells and Treg cells thus, creating a therapeutic vulnerability that may be exploited by ICBs to harness anti-tumour immunity. Furthermore, early- and late-stage ACM upregulated PD-L1 on the surface of Treg cells. Of particular note, late-stage ACM had a more substantial effect in upregulating PD-L1 on the surface of Treg cells compared with early-stage ACM, perhaps suggesting that ACM from late-stage OGJ patients possesses more immunoinhibitory effects compared with early-stage ACM. These findings highlight that blockade of PD-L1 and CTLA-4 may be a more rational therapeutic strategy than combining CTLA-4 blockade with PD-1 blockade, particularly when ACM downregulated PD-1 on the surface of T cells and specific Th1-like and Treg subsets.

## Supplementary Information

Below is the link to the electronic supplementary material.

Supplementary file1 (DOCX 786 KB)

## Data Availability

No datasets were generated from this study and clinical data cannot be made available.
