# Supplementary material for: Visceral adipose tissue secretome from early and late-stage oesophageal cancer patients differentially affects effector and regulatory T cells
Source: J Cancer Res Clin Oncol. 2023 Feb 15;149(9):6583–99. doi: 10.1007/s00432-023-04620-6 (PMC10356656; doi:10.1007/s00432-023-04620-6)
Supplement: Supplementary file 1 — Supplementary file1 (DOCX 786 KB) [file 432_2023_4620_MOESM1_ESM.docx]

Supplemental

**Figure S1: TNF-α was significantly decreased in ACM derived from late-stage OGJ patients compared with early-stage OGJ patients.** ACM from OGJ patients with early-stage tumours (pathological staging 0-II, n=8) versus late-stage tumours (pathological staging III-IV, n=10) was screened for a panel of pro-inflammatory mediators, pro-angiogenic mediators and immunomodulatory cytokines using multi-plex ELISA. Mann-Whitney test, *p≤0.05. Only mediators shown that were significantly different (or bordering significance) between OGJ patients with early vs. late-stage ACM are shown. Data was normalised to pg/g.

**Figure S2: There was no significant difference in the levels of a range of pro-inflammatory mediators, pro-angiogenic mediators and immunomodulatory cytokines in the ACM from OGJ patients with early versus late-stage tumours.** ACM from OGJ patients with early-stage tumours (pathological staging 0-II, n=8) versus late-stage tumours (pathological staging III-IV, n=10) was screened for a panel of pro-inflammatory mediators, pro-angiogenic mediators and immunomodulatory cytokines using multi-plex ELISA. Mann-Whitney test. Only mediators shown that were not significantly between OGJ patients with early vs. late-stage ACM. Mann-Whitney test, data was normalised to pg/g of tissue.

**Table S1. Patient Demographic Table**

| Table I |  |
| --- | --- |
| Patient Demographic Table. |  |
| Mean age (years) | 63.9 |
| Sex ratio (M:F) | 25:6 |
| Diagnosis (no. patients) |  |
| Pathological tumour stage (no. patients) |  |
| T0 | 3 |
| T1 | 7 |
| T2 | 2 |
| T3 | 19 |
| T4 | 0 |
| Pathological nodal status (no. patients) |  |
| Positive | 12 |
| Negative | 19 |
| Mean BMI (kg/m^2^) | 29.9 |
| Underweight (BMI < 20) | 0 |
| Normal weight (BMI 20–24.9) | 5 |
| Overweight (BMI 25–29.9) | 6 |
| Obese (BMI >30) | 20 |
| Mean visceral fat area (VFA) (cm^2^) (range) | 179.75 (52.25–369.26) |
